# Extracellular vesicles from human umbilical cord mesenchymal stem cells improve nerve regeneration after sciatic nerve transection in rats

**DOI:** 10.1111/jcmm.14190

**Published:** 2019-02-17

**Authors:** Yongbin Ma, Liyang Dong, Dan Zhou, Li Li, Wenzhe Zhang, Yu Zhen, Ting Wang, Jianhua Su, Deyu Chen, Chaoming Mao, Xuefeng Wang

**Affiliations:** ^1^ Department of Central Laboratory The Affiliated Hospital of Jiangsu University Zhenjiang China; ^2^ Department of Neurology Laboratory Jintan Hospital, Jiangsu University Jintan China; ^3^ Department of Nuclear Medicine and Institute of Oncology The Affiliated Hospital of Jiangsu University Zhenjiang China

**Keywords:** extracellular vesicles, human umbilical cord mesenchymal stem cell, improve, nerve regeneration, sciatic nerve transection

## Abstract

Peripheral nerve injury results in limited nerve regeneration and severe functional impairment. Mesenchymal stem cells (MSCs) are a remarkable tool for peripheral nerve regeneration. The involvement of human umbilical cord MSC‐derived extracellular vesicles (hUCMSC‐EVs) in peripheral nerve regeneration, however, remains unknown. In this study, we evaluated functional recovery and nerve regeneration in rats that received hUCMSC‐EV treatment after nerve transection. We observed that hUCMSC‐EV treatment promoted the recovery of motor function and the regeneration of axons; increased the sciatic functional index; resulted in the generation of numerous axons and of several Schwann cells that surrounded individual axons; and attenuated the atrophy of the gastrocnemius muscle. hUCMSC‐EVs aggregated to rat nerve defects, down‐regulated interleukin (IL)‐6 and IL‐1β, up‐regulated IL‐10 and modulated inflammation in the injured nerve. These effects likely contributed to the promotion of nerve regeneration. Our findings indicate that hUCMSC‐EVs can improve functional recovery and nerve regeneration by providing a favourable microenvironment for nerve regeneration. Thus, hUCMSC‐EVs have considerable potential for application in the treatment of peripheral nerve injury.

## 1 INTRODUCTION

Peripheral nerve injury often leads to limited nerve regeneration or incomplete functional recovery. In the field of regenerative medicine, mesenchymal stem (stromal) cells (MSCs) are considered as cell reservoirs given their ability to differentiate into specialized cells. MSCs can also secrete bioactive molecules that help construct the regenerative microenvironment after injury.^1^ Given these characteristics, MSCs have been proposed as a promising tool for nerve injury repair and regeneration.^2^ The regenerative potential of MSC therapies is mediated by paracrine actions.^3^ The paracrine effects exerted by human umbilical cord MSCs (hUCMSCs) are more intense than those exerted by bone marrow stem cells (BMSCs) and adipose‐derived MSCs.^4^ Accumulating evidence suggests that hUCMSCs promote axonal regeneration after peripheral nerve injury.^5^, ^6^, ^7^ Guo et al reported that the transplantation of hUCMSCs enhances axonal regeneration and functional recovery in peripheral nerve repair via paracrine action.^8^ Furthermore, hUCMSCs can reduce allogeneic or xenogeneic immune responses in graft animal models through their immunosuppressive ability.^9^, ^10^ Thus, hUCMSCs have a remarkable role in regenerative medicine by secreting soluble factors, which enhance and assist tissue repair, via a paracrine mechanism.

Extracellular vesicles (EVs) have been recently identified as one of the paracrine mechanisms of MSCs. EVs, which include exosomes, microvesicles and apoptotic bodies, are nanosized membrane fragments secreted by various types of cells, such as stem cells.^11^ EVs deliver a variety of proteins, lipids, cytokines, transcription factors and other biological materials to target cells.^12^ They are resistant to freeze‐thaw processes, can avoid the issues associated with MSC transplantation, and may be utilized in allogenic therapy because of their low immunogenicity.^13^ MSC‐derived EVs can be easily stored, exhibit superior safety and have remarkably promising applications in regenerative medicine.^3^ Recent studies on animal models have reported that EVs exhibit massive potential as cell‐free therapies. MSC‐EV treatment enhanced functional recovery and promoted neurite remodelling, neurogenesis and angiogenesis in rats with ischaemic stroke.^14^ Treatment with EVs derived from human BMSCs improved post‐stroke neuroregeneration and prevented postischaemic immunosuppression in mice.^15^ Our previous studies showed that BMSC‐derived EVs promoted nerve regeneration in rats with sciatic nerve crush injury.^16^ However, the effect of hUCMSC‐EVs on sciatic nerve transection remains to be examined.

In this study, we constructed a rat model of sciatic nerve transection. We observed that hUCMSC‐EVs improved motor functional recovery and defective nerve regeneration and attenuated denervated muscle atrophy. hUCMSC‐EVs can aggregate to nerve defects, down‐regulate pro‐inflammatory cytokines (interleukin [IL]‐6 and IL‐1β) and up‐regulate anti‐inflammatory cytokines (IL‐10). These effects indicate that hUCMSC‐EVs may contribute to neuroregeneration in rats with sciatic nerve defects. Thus, hUCMSC‐EVs represent a highly promising strategy for nerve regeneration after nerve transection and for peripheral nerve injury treatment.

## 2 MATERIALS AND METHODS

### 2.1 Animals

Male Sprague‐Dawley (SD) rats aged 3‐4 weeks old with body weights of 220‐230 g were purchased and maintained at the animal experimental centre of Jiangsu University in China. All animal experiments were approved by the Institutional Animal Care and Use Committee of Jiangsu University (Permit Number: JSU 16‐106).

### 2.2 Isolation and characterization of hUCMSCs

Human umbilical cord MSCs (hUCMSCs) were isolated and cultured in accordance with a previously described method.^17^ Fresh umbilical cords were obtained from consenting mothers at the People's Hospital of Jintan (Jintan, China) with the approval of the Jintan People's Hospital Ethics Committee. hUCMSCs from passages 3 to 5 were used for the experiments performed in this study. Alizarin Red and Oil Red O staining were performed as previously described. These tests revealed that hUCMSCs have osteogenic and adipogenic differentiation potential.^17^

Phycoerythrin (PE)‐conjugated CD14, CD19, CD34, CD45, CD73 and CD90 antibodies (all from BD Biosciences Pharmingen, San Jose, CA) were used to detect the typical surface markers of hUCMSCs from different passages through flow cytometry. Mouse PE‐IgG isotypic immunoglobulin was used as the negative control (NC). Cells were analysed by using FACSVerse^™^ instrument (BD Bioscience) with FlowJo software (Tree Star, Ashland, OR).

### 2.3 Isolation, purification and identification of hUCMSC‐EVs

Human umbilical cord MSCs (hUCMSCs) were cultured until 80%‐90% confluent. The supernatants of the cultures were collected through ultracentrifugation as previously described for the isolation of EVs.^17^ A bicinchoninic acid assay (BCA) protein assay kit was used to determine the protein content of EVs (Beyotime, Nantong, China). The NanoSight LM10 system (NanoSight, Amesbury, UK) was used to assess the particle size distribution of hUCMSC‐EVs. A transmission electron microscope (TEM) was used to identify the morphology of hUCMSC‐EVs as previously described.^17^

Filtered EVs were resuspended in PBS, incubated and stained with 20 μL of the directly fluorescent antibody CD63 (BD Biosciences) for the detection of the typical surface markers of hUCMSC‐EVs. Nonstained EVs were used as NCs. The phenotype of EVs was analysed by using BD Accuri C6 flow cytometer (Becton Dickinson, San Jose, CA, USA).

### 2.4 Surgical procedures and hUCMSC‐EV treatment

Rats were anaesthetized with 10 mg/kg xylazine and 75 mg/kg ketamine through intraperitoneal administration. Next, their left sciatic nerves were exposed and removed (3 mm). Nerve retraction resulted in the formation of a gap with a length of 5 mm. The proximal and distal stumps were fixed continuously with a simple 10‐0 nylon direct suture through the nerve epineurium and a silicone rubber tube (Helix Medical, Inc, Carpinteria, CA, USA). The distal and proximal nerve stumps were inserted 1 mm deep into the tube. A 5 mm‐long gap was maintained. The fascia and muscle layers were closed with 4‐0 nylon sutures. The skin was closed with a continuous running suture. After 24 hours of induction of the animal model, 100 μg of hUCMSC‐EVs (100 μL) in 0.2 mL of PBS or 0.2 mL of PBS was injected into the tail veins of the rats. Forty‐eight male SD rats were randomly allocated into the hUCMSC‐EV group (rats that received hUCMSC‐EV injection, n = 24) or control group (rats that received PBS injection, n = 24). Three rats that did not receive surgery or treatment were designated as normal control.

### 2.5 Functional assessment

Walking track analysis was performed prior to surgery and at 2, 4, 6 and 8 weeks post‐neurorrhaphy in accordance with a previously described protocol.^18^ Briefly, the hind paws of the rats were dipped in black ink. The rats were then allowed to demonstrate a standard walking trace on a strip of graph paper (30 × 7 cm). Sciatic functional index (SFI) was calculated on the basis of the following formula: SFI = 118.9 × (ETS − NTS)/NTS − 51.2 × (EPL − NPL)/NPL − 7.5. In this formula, E and N represent experimental and normal, respectively; ETS indicates the first to the fifth toes of experimental rats; NTS represents the normal toe spread; EPL signifies the operated experimental paw length; and NPL indicates the normal paw length. In general, 0 corresponds to normal function, and −100 corresponds to the complete loss of function.

### 2.6 Muscle weight measurement

The rats were killed at 8 weeks after surgery. Their gastrocnemius muscles were dissected and then weighed by using an electronic balance. The wet weight ratio of the gastrocnemius muscles was determined by using the following equation: operation side/normal side ×100%.

### 2.7 Haematoxylin and eosin staining

The nerve conduits and gastrocnemius muscles of the rats were harvested, fixed in 4% PFA at 4°C and embedded in paraffin. Longitudinal sections (12 μm in thickness) and cross‐sections (4 μm in thickness) of the middle portions of the grafts were prepared and subjected to haematoxylin and eosin (H&E) staining.

### 2.8 Immunohistochemical analysis

Nerve tissue sections were subjected to antigen retrieval at 3 days after injury. Slides were blocked and incubated with either a 1:200 dilution of monoclonal anti‐IL‐6 antibody (Servicebio, Wuhan, China), a 1:500 dilution of monoclonal anti‐IL‐1β antibody (Servicebio, Wuhan, China) or a 1:100 dilution of monoclonal anti‐IL‐10 antibody (Proteintech Group, Chicago, USA). Next, the slides were incubated with a 1:300 dilution of anti‐rat IgG‐horseradish peroxidase (Servicebio, Wuhan, China). Images were observed by using a Nikon Ti‐S microscope (Ti‐S, Nikon, Japan). The mean densities of IL‐6, IL‐1β and IL‐10 were determined by using Image‐Pro Plus software (Media Cybernetics, Bethesda, MD).

### 2.9 Transmission electron microscope

The mid‐points of repaired nerve tissues were resected at 4 weeks after surgery for TEM analysis. Tissue sections were prepared and observed through TEM (HT7700, Hitachi, Japan) as previously described.^19^ The number of myelin lamellae per field and the thickness of myelin sheaths were calculated.

### 2.10 Immunofluorescence

Immunofluorescence was used to determine nerve fibre morphology and myelin sheath regeneration. Nerve tissue paraffin sections were blocked and labelled with rabbit anti‐rat S‐100 (green) (Servicebio, Wuhan, China) (1:100 dilution), rabbit anti‐rat NF‐200 (red) (Servicebio, Wuhan, China) (1:100 dilution), rabbit anti‐rat myelin basic protein (MBP) (green) (Servicebio, Wuhan, China) (1:100 dilution) or mouse monoclonal anti‐BrdU (red) (Servicebio, Wuhan, China) (1:100 dilution). Alexa Fluor 488 goat anti‐rabbit antibody (Servicebio, Wuhan, China) (1:400 dilution) or Cy3 goat anti‐mouse antibody (Servicebio, Wuhan, China) (1:300 dilution) were used as the secondary antibody. Myelin regeneration was determined by counting the number of myelinated axons per field in 10 randomly selected sections of regenerative nerve tissue under ×400 magnification. DiR‐labelled EVs in the distal nerve stumps were identified through confocal microscopy analysis for the detection of the specific Schwann cell (SC) marker S‐100. Sections were blocked and labelled with rabbit anti‐rat S‐100 (Green) (Servicebio, Wuhan, China) (1:100 dilution), whereas Alexa Fluor 488 goat anti‐rabbit antibody (Servicebio, Wuhan, China) (1:400 dilution) was used as the secondary antibody.

### 2.11 Western blot analysis

At 4 weeks after surgery, the mid‐points of repaired nerve tissue were homogenized and subjected to protein extraction for Western blot analysis in accordance with a previously described procedure.^19^ Rabbit monoclonal antibody of myelin basic protein (MBP) (Servicebio, Wuhan, China) (1:400 dilution) and β‐actin (Cell Signaling Technology, Danvers, MA, USA) (1:1,000 dilution) were used as the primary antibodies. The second antibody was goat anti‐rabbit IgG (Servicebio, Wuhan, China) (1:1,000 dilution). β‐actin was used as an internal control.

### 2.12 Tracking of hUCMSC‐EVs

Human umbilical cord MSC‐derived extracellular vesicles (hUCMSC‐EV) suspensions were labelled with DiR (Invitrogen, Carlsbad, CA, USA), resuspended through ultracentrifugation to precipitate the solution and washed twice with PBS. Then, 100 μg of DiR‐labelled EVs were intravenously injected into the animal models. At 24 hours after injection, the rats were anaesthetized and prepared for in vivo imaging. The distributions of labelled EVs in rats were detected by using the IVIS Spectrum Imaging System (PerkinElmer).

### 2.13 Statistical analysis

The data are expressed as means ± standard error of the mean. Statistical analyses were performed with GraphPad Prism V 5.0 software (GraphPad, San Diego, CA, USA). The Student's *t* test was used to evaluate the significance and *P* *< *0.05 was regarded as statistically significant.

## 3 RESULTS

### 3.1 Typical characteristics of hUCMSCs and hUCMSC‐EVs

**Figure 1:**
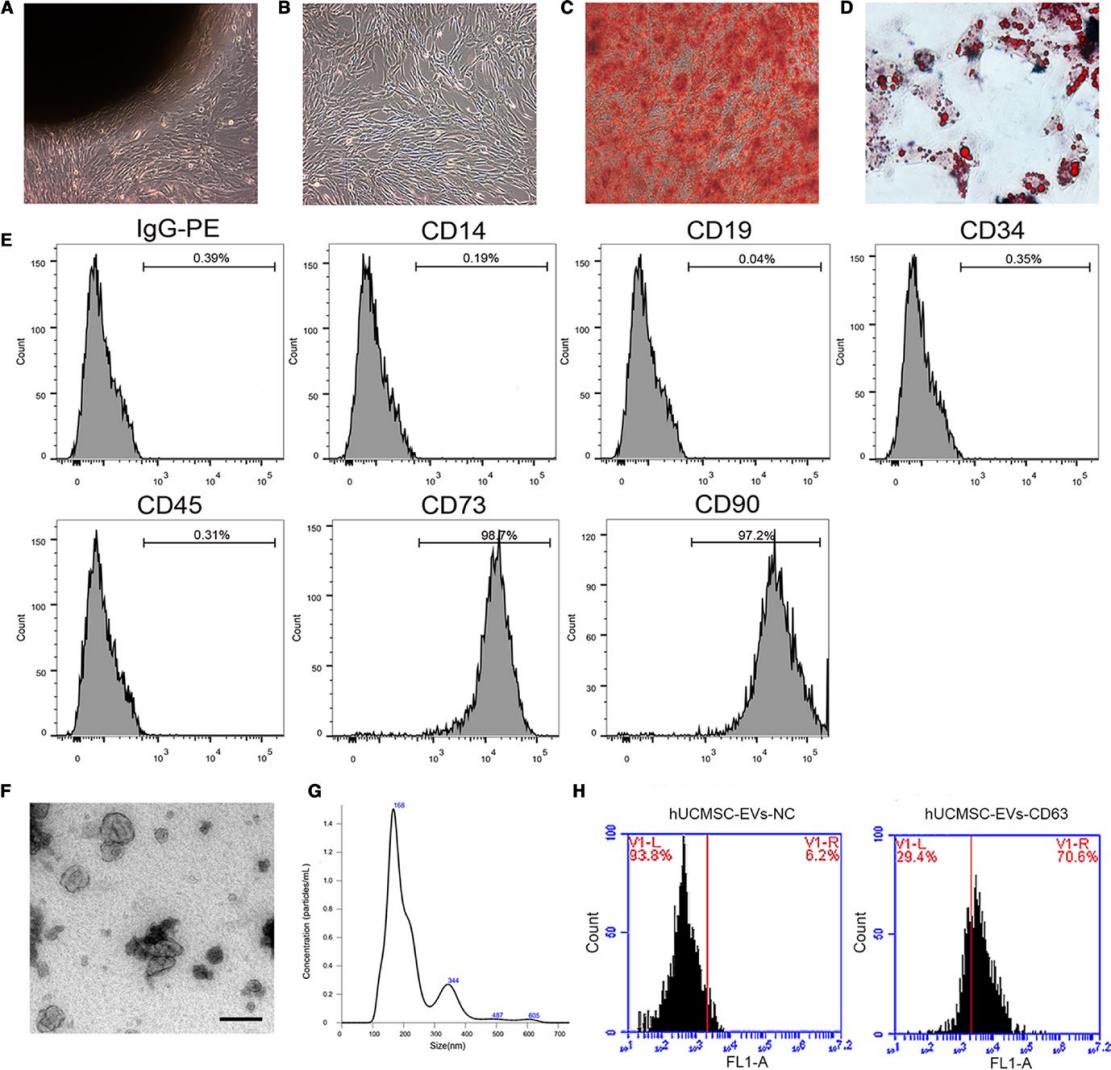
Identification of human umbilical cord MSCs (hUCMSCs) and human umbilical cord MSC‐derived extracellular vesicles (hUCMSC‐EVs). (A and B) Morphology of hUCMSCs (passages 0 and 3) under light microscopy (×100 magnification). (C and D) hUCMSCs induced for differentiation into osteocytes (×100) and adipocytes (×200). Cells stained with Alizarin Red and Oil red O. E, Results for the flow cytometry analyses of phenotypic markers related to hUCMSCs. F, Representative transmission electron microscope (TEM) image of purified hUCMSC‐EVs presenting a typical cup shape. The scale bar represents 100 nm. G, Particle sizes of hUCMSC‐EVs determined through nanoparticle tracking analysis. H, Flow cytometry results for CD63, a surface marker of hUCMSC‐EVs (hUCMSC‐EVs‐CD63). EVs reacted with the isotype antibody were applied as the negative control (hUCMSC‐EVs‐NC)

After 10 days of initial culture, adherent cells displayed long spindle‐like shapes, formed colonies and reached confluency (Figure 1A,B). The MSCs showed multilineage potential to differentiate into osteocytes and adipocytes, as indicated by positive Alizarin Red (Figure 1C) and Oil Red O (Figure 1D) staining. Fluorescence‐activated cell sorting demonstrated that the cells were positive for CD73 and CD90, but negative for CD14, CD19, CD34 and CD45 (Figure 1E). These data indicate that we had efficiently generated hUCMSCs, as confirmed on the basis of the criteria defined by the International Society for Cellular Therapy.^20^

Isolated and purified EVs were assessed through TEM, nanoparticle tracking analysis (NTA) and flow cytometry. TEM revealed that the hUCMSC‐EVs were round‐shaped membrane particles with a typical cup shape (Figure 1F). The diameters of hUCMSC‐EVs ranged from 80 to 650 nm with an average of 168 nm as recorded by NTA (Figure 1G). Flow cytometry analysis revealed that the majority of hUCMSC‐EVs expressed the specific marker CD63, which is a representative marker of EVs (Figure 1H). As noted above, hUCMSCs and their corresponding EVs were successfully isolated and characterized.

### 3.2 hUCMSC‐EV treatment improved the functional recovery of the sciatic nerve

**Figure 2:**
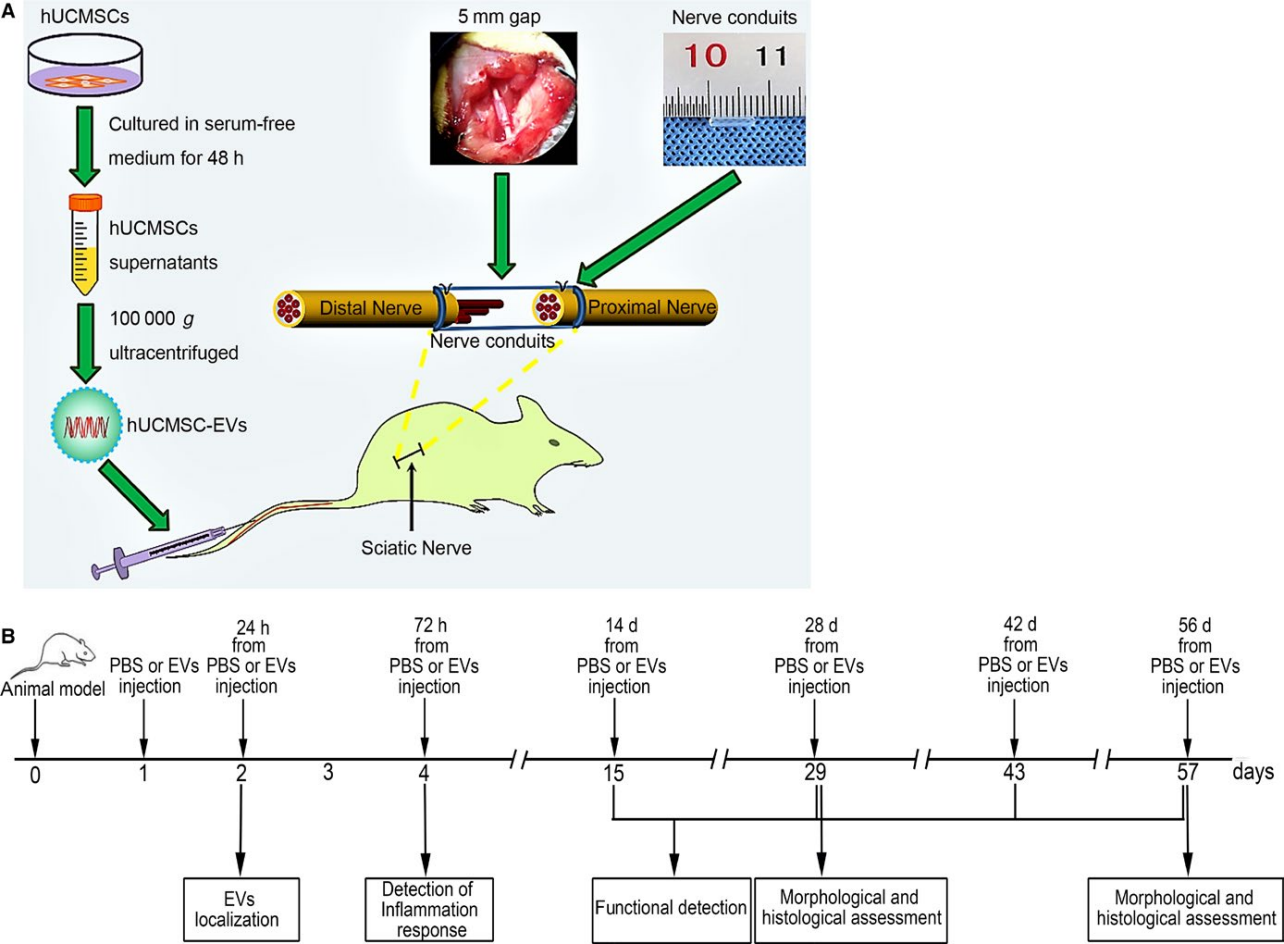
Experimental scheme. A, Rat model construction and hUCMSC‐EV collection and treatment. hUCMSCs were cultured in a 10 cm dish containing serum‐free medium for 48 h. Then, the supernatant was extracted, and EVs were collected through ultracentrifugation. Nerve retraction after surgical transection resulted in the formation of a 5‐mm long gap in the left sciatic nerve. EV or PBS solutions were injected into the tail veins of rats at 24 h post‐surgery. B, Schematic representation of hUCMSC‐EV or PBS treatment after sciatic nerve transection. Rats underwent sciatic nerve transection surgery on day 0 and received hUCMSC‐EV or PBS treatment on day 1. hUCMSC‐EV localization was determined on day 2. The inflammatory response of the distal nerve stump was detected on day 4. Rat motor function was assessed on days 15, 29, 43 and 57. Nerve regeneration was determined on day 29 and 57 through morphological and histological analyses

**Figure 3:**
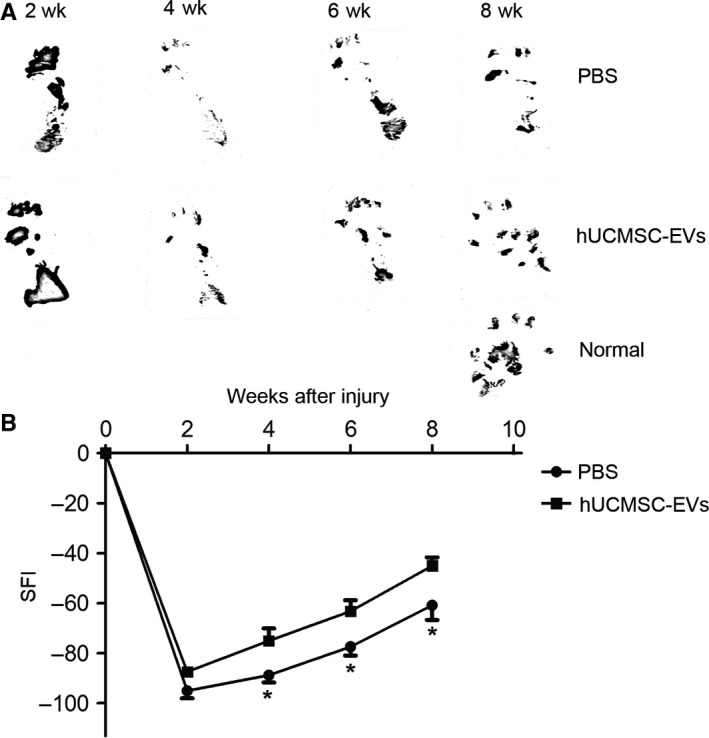
Human umbilical cord MSC‐derived extracellular vesicles (hUCMSC‐EVs) improved functional recovery after sciatic nerve transection. Motor function at 2, 4, 6 and 8 wk post‐neurorrhaphy was analysed on the basis of the walking track test. A, Representative pawprint patterns from rats treated with PBS or hUCMSC‐EVs. B, Quantification of the SFI of PBS‐ and hUCMSC‐EV‐treated rats. n = 12. Data are presented as means ± standard error of the mean (SEM). *P < 0.05

We constructed a rat model of sciatic nerve transection to examine the effects of hUCMSC‐EVs on sciatic nerve defects. Figure 2A illustrates the construction of the rat model and the collection and the treatment of hUCMSC‐EVs. Figure 2B shows a schematic of the experimental process after hUCMSC‐EV or PBS treatment. Walking track analysis was used to assess the motor function recovery of rats. SFI was used to reveal the degrees of improvement exhibited by the hUCMSC‐EV and control groups. The results of walking track analysis shown in Figure 3A,B indicate that the PBS group demonstrated neurological functional recovery and that the hUCMSC‐EV treatment group showed improved functional recovery. At 8 weeks after sciatic nerve transection, the walking track patterns of the hUCMSC‐EV‐treated rats were almost similar to those of the normal rats. The SFI scores for the hUCMSC‐EV group drastically increased relative to those of the control group at 4, 6 and 8 weeks after surgery. These results indicate that treatment with hUCMSC‐EVs improved the motor function recovery of the severed sciatic nerve.

### 3.3 Morphological analysis of nerve regeneration

**Figure 4:**
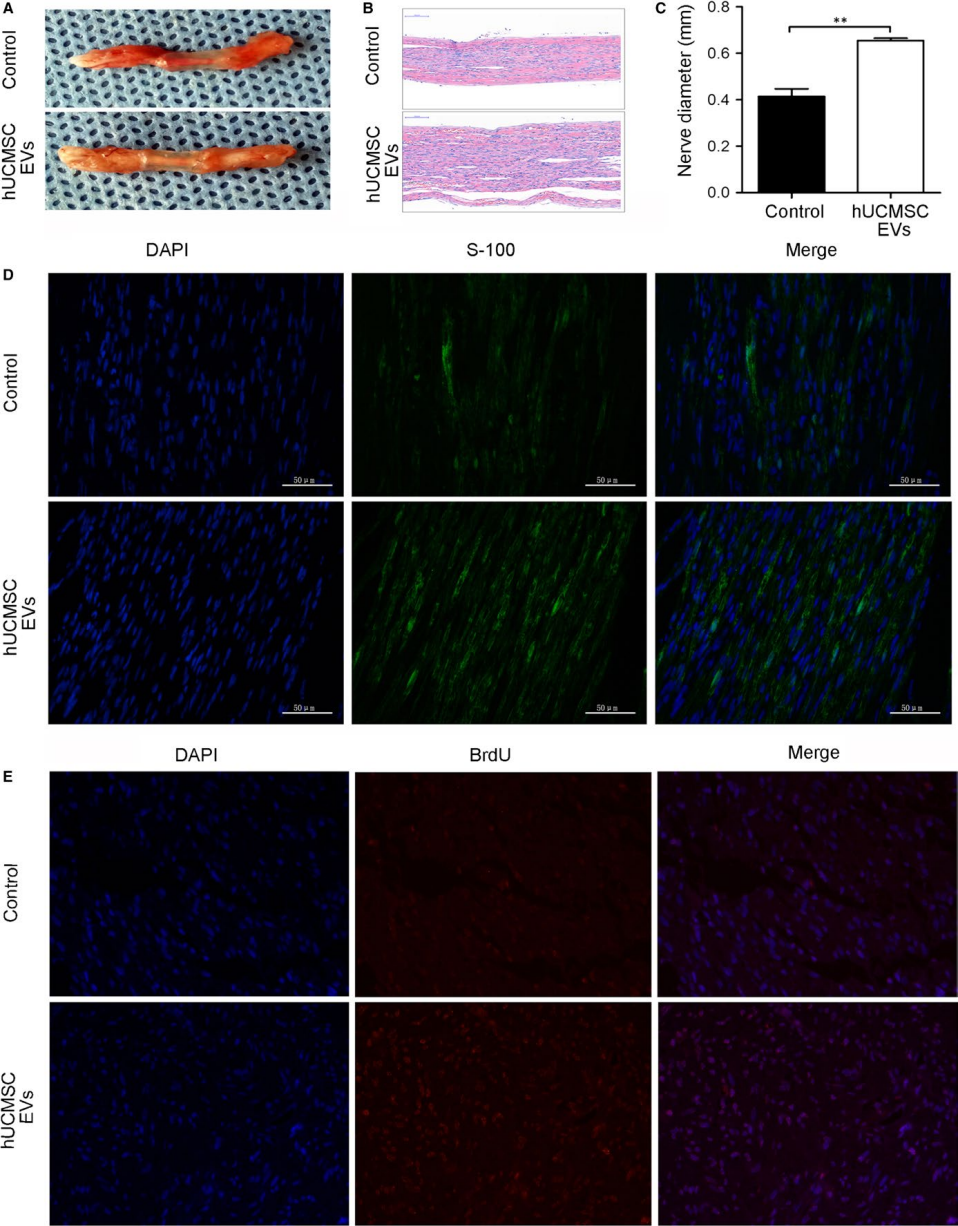
Human umbilical cord MSC‐derived extracellular vesicles (hUCMSC‐EVs) promoted the morphological recovery of regenerated nerves 8 wk after sciatic nerve transection. A, Harvest of nerve conduits. B, H&E staining of regenerated nerve tissue. C, Diameters of regenerated nerves from rats that received different treatments. D, Representative image of S‐100‐positive fibres (green) from the rat model of sciatic nerve injury. E, Representative image of BrdU‐positive fibres (red) from the rat model of sciatic nerve injury. Nuclei were stained with 4ʹ,6ʹ‐diamidino‐2‐phenylindole (blue). The scale bar represents 50 μm. n = 12. Data are presented as means ± SEM. **P < 0.01

We observed the morphology of regenerating nerves in middle segments at 8 weeks after sciatic nerve transection. The conduit was completely harvested, and two stumps in resected median nerves were connected. The results showed that the hUCMSC‐EV and the control groups exhibited nerve generation. The regenerated nerves of rats in the experimental group were larger than those of rats in the control group (Figure 4A). Specifically, H&E staining revealed that the diameters of the regenerated nerve fibres in the hUCMSC‐EV group were significantly greater than those in the control group (Figure 4B,C) (*P < *0.05). S‐100 is a surface marker of SCs, which wrap nerve fibres to form myelin sheaths.^21^ Immunofluorescence staining revealed that S‐100‐positive fibres had appeared in the hUCMSC‐EV and control groups. However, the immunofluorescence density of S‐100‐positive fibres in the hUCMSC‐EV group was higher than that in the control group (Figure 4D). The results of BrdU staining showed that hUCMSC‐EV treatment promoted Schwann cell proliferation (Figure 4E). The axonal regeneration exhibited by the hUCMSC‐EV treatment group was better than that exhibited by the control group at 8 weeks after surgery. This result suggests that hUCMSC‐EVs promoted injured sciatic nerve remyelination and axonal regeneration.

### 3.4 Human umbilical cord MSC‐derived extracellular vesicles increased the total number of myelinated fibres

**Figure 5:**
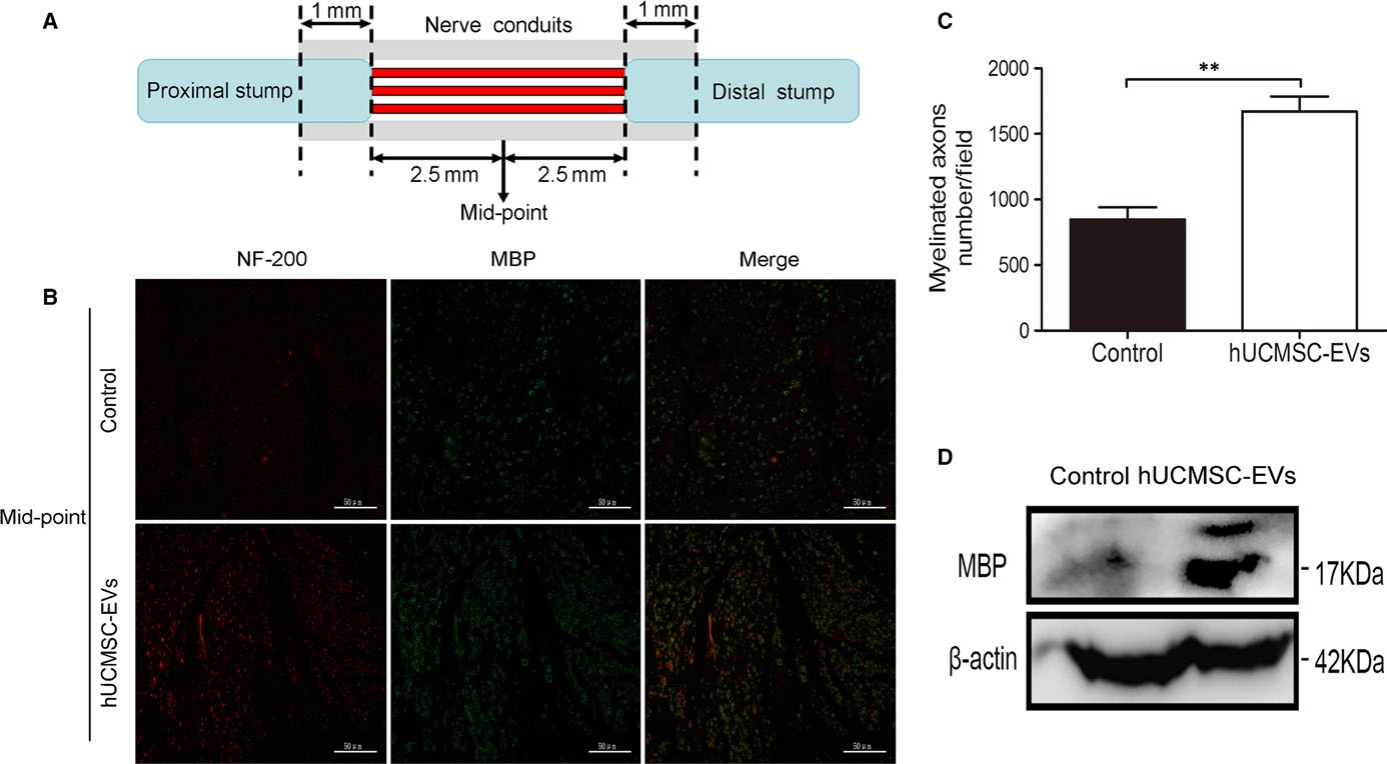
Human umbilical cord MSC‐derived extracellular vesicles (hUCMSC‐EVs) facilitated axonal regeneration after sciatic nerve injury. A, Transverse sections obtained at the mid‐points of repair sites. B, Images of NF‐200 and MBP antibody staining for the detection of regenerated axons in the conduit under ×400 magnification. The scale bar represents 50 μm. C, Quantification of myelinated neurons. n = 12. Data are presented as means ± SEM. **P < 0.01. D, MBP protein expression quantified through Western blot analysis. A representative blot from two groups is shown

**Figure 6:**
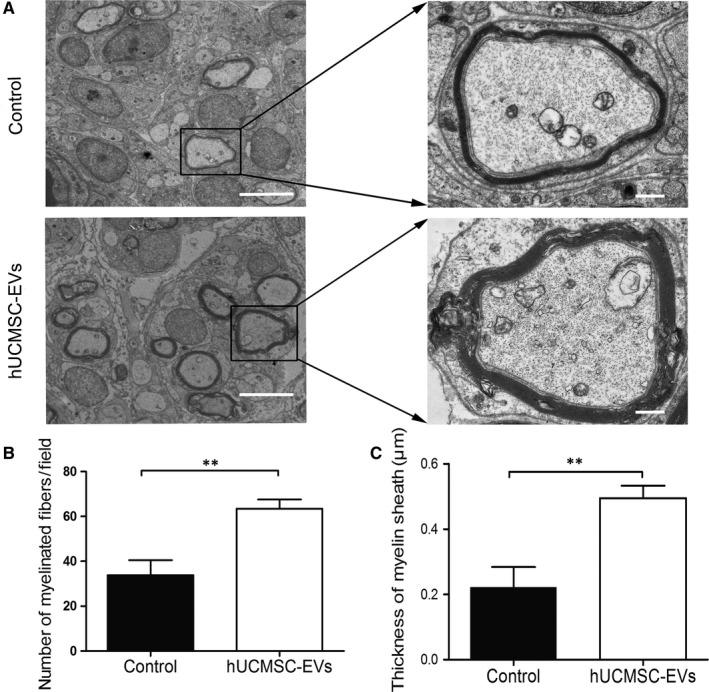
Human umbilical cord MSC‐derived extracellular vesicles (hUCMSC‐EVs) promoted the myelination of regenerating axons after sciatic nerve injury. TEM images of regenerating nerves in the mid‐points of repair sites at 4 wk after surgery. A, Representative TEM image of the two groups. The left‐hand scale bar represents 20 μm, and the right‐hand scale bar represents 2 μm. B, Number of myelinated fibres per field and (C) thickness of regenerated myelin sheaths. n = 12, Data are presented as means ± SEM. **P < 0.01

The mid‐points of transverse sections were obtained for the detection of myelin regeneration through immunofluorescence analysis. Figure 5A illustrates the mid‐point of the repaired nerve tissue. At 8 weeks after surgery, we performed immunostaining to detect the presence of the axon marker NF‐200 and the SC marker MBP in the cross‐sections of regenerated sciatic nerves. As shown in Figure 5B, the fluorescent signals of NF‐200 and MBP were predominantly detected in sections from the hUCMSC‐EV group, whereas minimal or no signals were detected in sections from the control group. Quantifying the myelinated axons of the sciatic nerve revealed that hUCMSC‐EV treatment increased axonal myelination at 8 weeks after axotomy (Figure 5C). Furthermore, at 4 weeks after surgery, MBP expression was up‐regulated under the hUCMSC‐EV treatment relative to that under the PBS control treatment (Figure 5D). TEM revealed that the numbers of myelinated fibres and the thicknesses of myelin sheaths in hUCMSC‐EV‐treated rats were higher than those in PBS‐treated rats (Figure 6). These findings indicate that hUCMSC‐EV treatment resulted in the generation of a high number of axons and several SCs around individual axons and suggest that hUCMSC‐EVs may facilitate the myelination of regenerating axons.

### 3.5 Human umbilical cord MSC‐derived extracellular vesicles reduced the degree of gastrocnemius muscle atrophy

**Figure 7:**
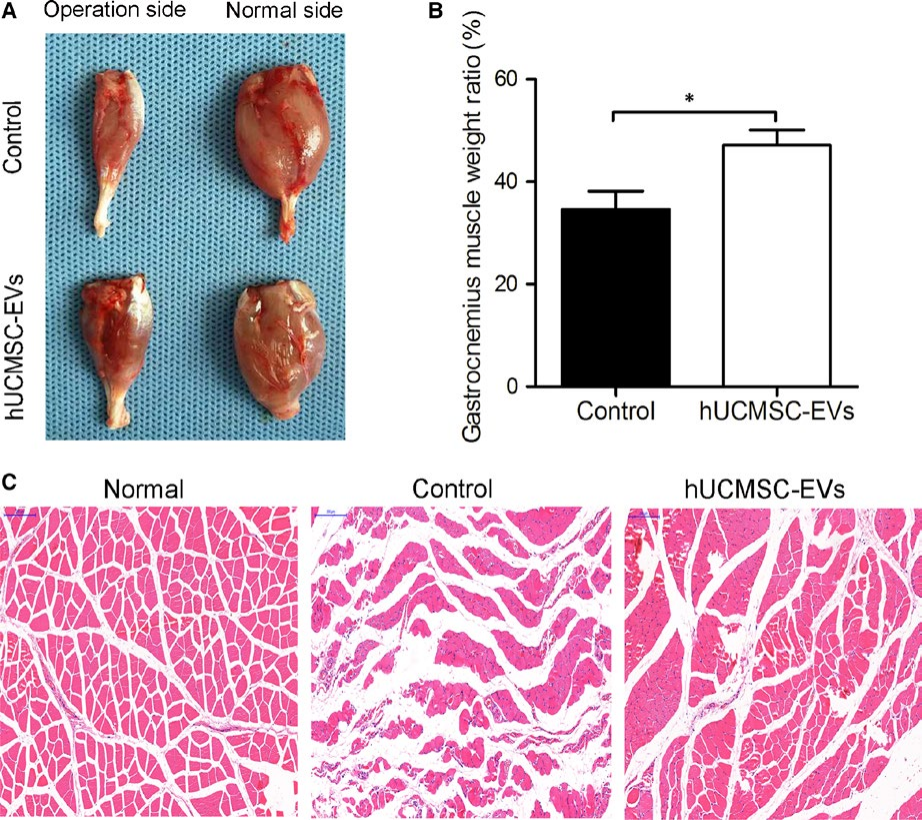
Human umbilical cord MSC‐derived extracellular vesicles (hUCMSC‐EVs) attenuated the atrophy of gastrocnemius muscles. A, Morphology of gastrocnemius muscles collected from hUCMSC‐EV or PBS (control) groups at 8 wk after sciatic nerve transection. B, Gastrocnemius muscle weight ratio (operation sides/normal sides) of rats under different treatments. C, H&E staining of gastrocnemius muscles. The scale bar represents 200 μm. n = 12. Data are presented as means ± SEM. *P < 0.05

The gastrocnemius muscle loses mass after sciatic nerve transection because it fails to receive neural innervation. We weighed gastrocnemius muscles at 8 weeks to evaluate muscle innervation recovery. As shown in Figure 7A, the wet weights of the gastrocnemius muscles from the hUCMSC‐EV group were higher than those from the control group. In addition, the wet weight ratio of the gastrocnemius muscles of the hUCMSC‐EV group was higher than that of the control group (Figure 7B). These results suggest that hUCMSC‐EV treatment resulted in the extensive innervation of the gastrocnemius muscles. H&E staining showed that the atrophy of gastrocnemius muscle fibres was attenuated in the hUCMSC‐EV group. The morphology of gastrocnemius muscle fibres in the hUCMSC‐EV group was similar to that of normal muscle. However, the reduced area of the gastrocnemius muscle fibres in the control group was indicative of severe gastrocnemius muscle atrophy (Figure 7C).

### 3.6 Human umbilical cord MSC‐derived extracellular vesicles aggregated to rat nerve defects and modulated their inflammatory responses

**Figure 8:**
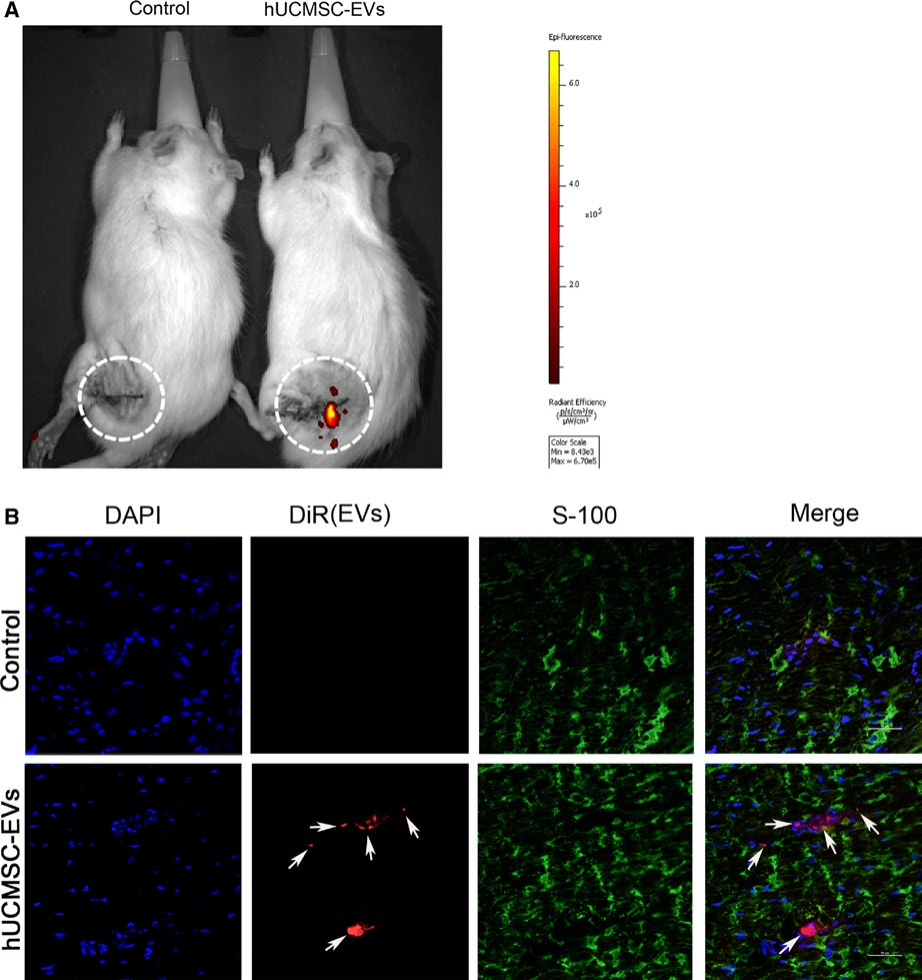
Tracing of DiR‐labelled hUCMSC‐EVs in vivo. A, DiR‐labelled EV (red) or PBS solutions were injected into the rat tail vein for 24 h. A representative in vivo image acquired with the IVIS imaging system is presented. The fluorescence intensity scale is indicated on the right side of the images. B, SC‐labelled S‐100 and DiR‐labelled EVs were evaluated in the distal nerve stump at 24 h after EV injection. Representative microphotographs were obtained through confocal microscopy. Arrows indicate DiR‐labelled EVs. The scale bar represents 50 μm. n = 3

**Figure 9:**
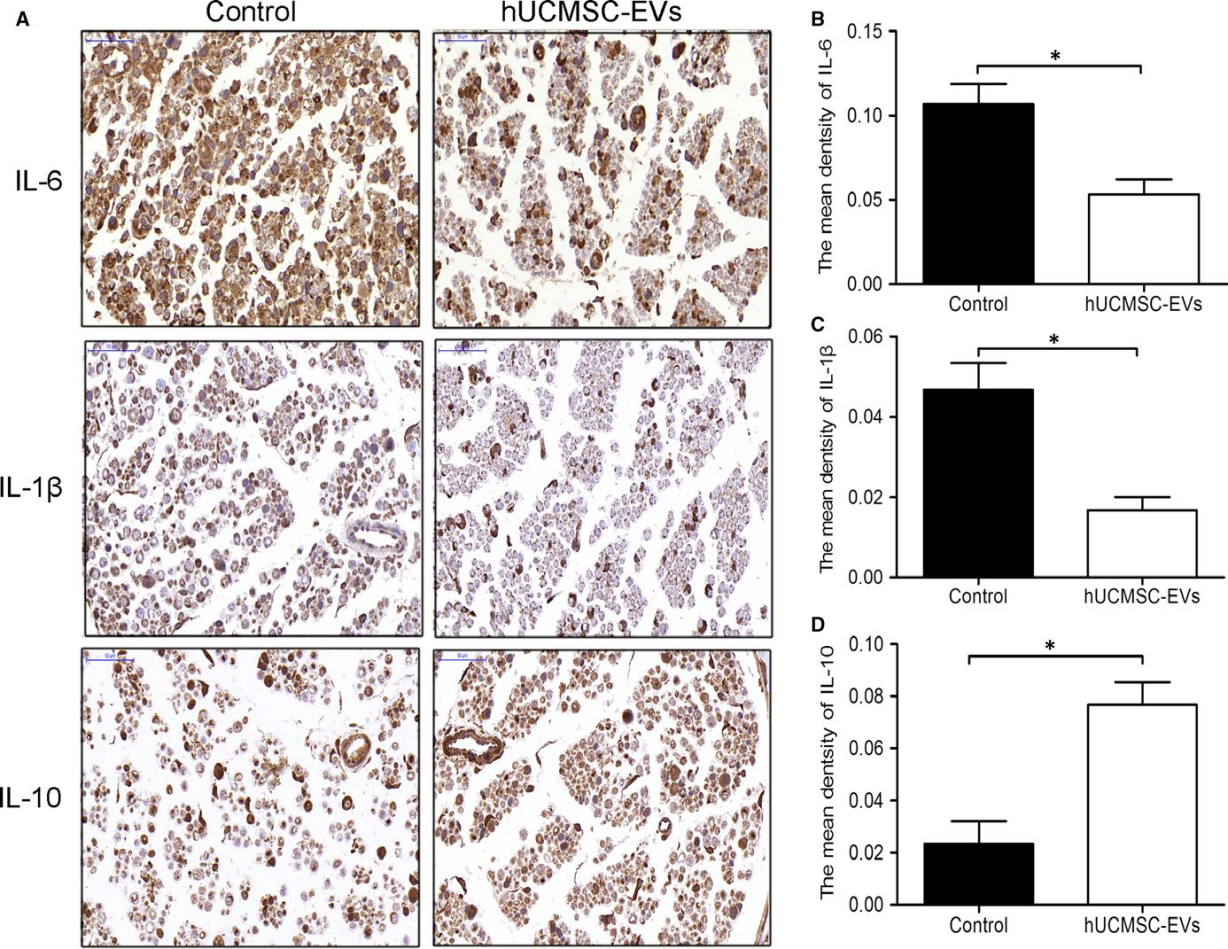
Human umbilical cord MSC‐derived extracellular vesicles (hUCMSC‐EVs) modulated inflammation in the distal nerve stump. A, Longitudinal sections of the regenerated nerves were stained with IL‐6, IL‐1β and IL‐10 antibodies at 3 d after injury. IL‐6‐, IL‐1β‐ and IL‐10‐positive sites were stained brown with an immunohistochemical stain. The scale bar represents 50 μm. B, Quantitative analyses of IL‐6‐, IL‐1β (C) and IL‐10‐positive areas in each group (D). n = 3. Data are presented as means ± SEM, *P < 0.05

Mesenchymal stem cells (MSCs) can home and engraft to injured tissues.^13^ Thus, we investigated whether MSC‐derived EVs feature a similar homing function. We assessed the biodistribution of EVs in rats through fluorescent imaging by using the IVIS Lumina II system. As shown in Figure 8A, DiR‐labelled EVs from hUCMSC aggregated to nerve defects at 24 hours after injection into rat tail veins. The rats were then killed immediately, and nerves distal to the nerve defects were harvested for tissue sectioning. The immunofluorescence staining results of longitudinal sections revealed that DiR‐labelled EVs reached the distal ends of the nerve defects (Figure 8B). Given the evidence demonstrating that MSC‐EVs limited the postischaemic inflammation that contributes to the development of ischaemic brain injury,^15^ we also investigated whether nerve injury‐induced immune responses were regulated by hUCMSC‐EVs. The distal nerve stump was subjected to histochemical staining at 3 days after surgery. As shown in Figure 9, relative to those in the control group, pro‐inflammatory cytokines (IL‐6 and IL‐1β) were down‐regulated and anti‐inflammatory cytokines (IL‐10) were up‐regulated in the hUCMSC‐EV‐treated group (Figure 9B‐D). These results suggest that hUCMSC‐EVs can engraft to nerve defects and modulate their inflammatory responses. This effect may promote the regeneration of defective nerves.

## 4 DISCUSSION

Mesenchymal stem cells transplantation has been widely used in regenerative medicine to relieve nerve injury. Stem cells exert their reconstructive effects via a paracrine action mediated by EVs.^22^ Recent studies based on animal models have shown that EV‐based therapy has considerable potential because MSC‐derived EVs can simulate the pleiotropic functions, such as anti‐inflammatory, immunomodulatory, anti‐fibrotic and anti‐apoptotic actions, of their parent cells.^23^

In this study, we used a rat model of sciatic nerve transection injury to assess the effects of hUCMSC‐derived EVs. hUCMSCs are good choices for clinical applications because they can be easily obtained and proliferate in vitro. Furthermore, they possess low immunogenicity and high paracrine potential for promoting injury tissue repair.^24^, ^25^ However, the effectiveness of hUCMSC‐EVs in treating large nerve gaps had not been assessed. Here, we demonstrated that hUCMSC‐EVs effectively promoted motor function recovery and nerve regeneration in a rat model of sciatic nerve defects.

First, we identified the characteristics of hUCMSCs, which share a similar fibroblastoid‐like morphology, differentiation potential and marker expression with cells with a bone marrow origin.^26^ We successfully extracted hUCMSC‐EVs, which presented a cup‐shaped membrane structure when viewed through TEM. The sizes and shapes of the hUCMSC‐EVs that we obtained in the present study are consistent those of hUCMSC‐EVs that we collected in a previous work.^17^

Similar to the effects of MSCs on peripheral nerve injury,^26^, ^27^ the effects of hUCMSC‐EVs rapidly and drastically improved the motor function recovery of the sciatic nerve over time. These effects were proven by the walking track analysis results and SFI scores (Figure 3). These findings imply that hUCMSC‐EVs facilitate intensive neural regeneration. The regeneration of peripheral nerves is a complex process that involves the survival of injured nerves, the remyelination of regenerative axons, the reinnervation of the appropriate nerves and the recovery of the connection between regenerative axons and target organs.

SCs can enable the myelination of regenerating axons, which is a crucial step in the regeneration and functional recovery of injured peripheral nerves. S100, a marker of mature SCs, has been used to observe SC proliferation and myelin sheath formation.^28^ We observed that the sizes and diameters of regenerative nerves in the hUCMSC‐EV group were larger than those of regenerative nerves in the control group. S100 immunoreactivity and Brdu incorporation further confirmed that hUCMSC‐EV treatment improved nerve regeneration after sciatic nerve injury (Figure 4). NF200 is extensively distributed in myelinated axons in the nervous system,^29^ and MBP is constitutively expressed in the myelin sheath of oligodendrocytes and SCs.^30^ NF200 and MBP immunolabeling revealed that hUCMSC‐EVs effectively stimulated nerve regeneration and nerve fibre myelination, which are important indexes of axonal regeneration and myelin sheath formation, after rat sciatic nerve transection. TEM analysis further confirmed that hUCMSC‐EV treatment increased the number of myelinated fibres and the thicknesses of myelin sheaths and facilitated the myelination of regenerating axons.

The gastrocnemius muscle will recover from atrophy and regain mass after undergoing regenerating nerve reinnervation.^31^ In this study, we found that treatment with hUCMSC‐EVs dramatically attenuated muscular atrophy. The high mean gastrocnemius muscle weight ratios of the hUCMSC‐EV group provided indirect evidence for successful end‐organ reinnervation. This result suggests that hUCMSC‐EV treatment improved muscle activity and function. Walking track analysis is widely applied to evaluate the recovery of the motor function of nerve‐injured rats.^32^ hUCMSC‐EV‐treated animals displayed remarkable motor function recovery at 4 weeks after surgery. This result implies the early occurrence of neural regeneration. An advantageous regenerative microenvironment, which facilitates the regeneration of injured tissue, is established through the release of neurotrophic and growth‐related factors by stem cells.^33^ Furthermore, these cells can secrete vesicles, which can carry the soluble factors necessary for the treatment of peripheral nerve injury.^26^ This view is consistent with the results of the present study. The results of this study, in turn, are consistent with those of a previous work demonstrating that adipose MSC‐derived microvesicles enhance sciatic nerve regeneration in rats.^34^

Mesenchymal stem cells preferentially home to injury sites, where they assist and promote tissue regeneration.^35^ Although MSCs were originally thought to home to injury sites, MSC secretomes, including EVs, likely exert this homing and migration effect.^36^, ^37^ Nanosized EVs enable communication between MSCs and immune cells and disseminate their parental components throughout the body via biological fluids.^38^ Similar to MSCs, hUCMSC‐derived EVs can home to rat nerve injuries as revealed by fluorescent imaging (Figure 8). Although the precise molecular mechanisms through which MSC‐EVs migrate into sites of injury remain incompletely defined, their high expression of migration‐associated cytokines and chemokines^27^, ^39^ may account for their homing function.

Extracellular vesicles derived from MSCs can exert the anti‐inflammatory effects of their parent cells. EVs generated by MSCs modulate T cell responses and limit pro‐inflammatory responses in type I diabetes.^40^ The modulation of postischaemic peripheral and cerebral immune responses through MSC‐EV delivery improves post‐stroke neuroregeneration.^15^ Consistent with previously reported observations that MSC‐derived EVs function similarly to MSCs by decreasing inflammatory cytokines and increasing anti‐inflammatory responses,^41^ hUCMSC‐EV treatment down‐regulated the pro‐inflammatory cytokines IL‐6 and IL‐1β and up‐regulated the anti‐inflammatory cytokine IL‐10 at the distal ends of nerve defects. These results are consistent with previous findings showing that MSC‐EVs exhibit immunosuppression activity similar to that exhibited by MSCs by releasing IL‐10, increasing Treg populations and down‐regulating tumour necrosis factor‐α and IL‐1β.^42^ Numerous molecular signalling systems, such as Neuregulin‐1/ErbB signalling, play an important role in remyelination after injury. Neuregulin‐1/ErbB elicits the activation of the PI3K pathway, which results in the phosphorylation of Akt.^43^ Akt phosphorylation, in turn, results in the differentiation and axonal myelination of Schwann cells in injured peripheral nerves.^44^ Neuregulin‐1 promotes remyelination and induces a proregenerative inflammatory response by elevating IL‐10 in focal demyelinating spinal cord lesions.^45^ IL‐10 is a known proregenerative cytokine, and increases in IL‐10 levels are associated with wound healing, tissue remodelling and remyelination.^46^, ^47^ Thus, we suggested that hUCMSC‐EVs can aggregate to injured nerves to inhibit injury‐induced inflammation. In addition, similar to hUCMSCs, hUCMSC‐EVs secrete neurotrophic and nerve growth factors^48^, ^49^, ^50^ to facilitate the establishment of a favourable microenvironment for nerve regeneration. The mechanism through which hUCMSC‐EVs promote nerve regeneration requires further analysis.

The source of isolated MSCs may influence therapeutic efficiency. MSCs isolated from Wharton's jelly (WJ) in umbilical cords have a notable expansion capability, a high proliferation rate and strong immunomodulatory capacities.^26^ Ribeiro et al reported that adipose tissue MSCs (AT‐MSCs) and WJ‐MSCs can secrete neuroregulatory/trophic factors that can enhance the metabolic viability of hippocampal neurons in vitro. AT‐MSCs require the addition of exogenous factors, such as basic fibroblast growth factor, to primary cultures to increase the viability of hippocampal neurons, whereas WJ‐MSCs can promote neuronal survival without the addition of exogenous factors.^51^ Thus, EVs from hUCMSCs are safe and promising tools for clinical peripheral nerve repair. Nevertheless, additional evidence is needed to further characterize their proneural repair mechanisms.

In summary, we demonstrated that hUCMSC‐EVs effectively promoted functional recovery and nerve regeneration in a rat model of sciatic nerve defects. Our findings provide the possibility for a promising method of clinical peripheral nerve repair. The clinical application of hUCMSC‐EVs is more expedient than the administration of stem cells.

## CONFLICT OF INTEREST

The authors declare that they have no competing interests. The funding agencies played no role in the design or implementation of the study, analysis or interpretation of the data or the preparation and submission of the manuscript.

## AUTHORS’ CONTRIBUTIONS

Conceived and designed the experiments: YBM XFW. Performed the experiments: YBM LYD DZ LL. Analysed the data: WZZ YZ TW. Contributed reagents/materials/analysis tools: JHS DYC CMM. Wrote the paper: YBM XFW. All authors read and approved the final manuscript.
